# Influence of Sexually Transmitted Infections in Pregnant Adolescents on Preterm Birth and Chorioamnionitis

**DOI:** 10.1155/2020/1908392

**Published:** 2020-03-25

**Authors:** Esther Fuchs, Maggie Dwiggins, Erica Lokken, Jennifer A. Unger, Linda O. Eckert

**Affiliations:** ^1^Department of Obstetrics and Gynecology, University of Washington, USA; ^2^Department of Pediatric and Adolescent Gynecology, MedStar Washington Hospital Center, USA; ^3^Department of Epidemiology, University of Washington School of Public Health, USA; ^4^Departments of Obstetrics and Gynecology and Global Health, University of Washington, USA

## Abstract

**Background:**

Adolescents have an increased risk of preterm birth (PTB) and sexually transmitted infections (STIs). We examined the prevalence and impact of STIs (gonorrhea, chlamydia, and trichomonas) on PTB and chorioamnionitis in pregnant adolescents.

**Methods:**

This retrospective cohort study utilized the first pregnancy delivered at an urban hospital among patients ≤ 19 years old over a 5-year period. Poisson regression with robust standard errors was used to estimate prevalence ratios (PR) and 95% confidence intervals (CI) of the association between STIs and PTB (<37 weeks) and chorioamnionitis identified by clinical or placental pathology criteria.

**Results:**

739 deliveries were included. 18.8% (*n* = 139) of births were preterm. The overall prevalence of STIs during pregnancy was 16.5% (*Chlamydia trachomatis*: 13.1%, *n* = 97; *Trichomonas vaginalis*: 3.7%, *n* = 27; and *Neisseria gonorrheae*: 3.1%, *n* = 23). Detection of *C. trachomatis*, *T. vaginalis*, or *N. gonorrheae* was not associated with increased PTB. While infection with *N. gonorrheae* and *C. trachomatis* did not increase the likelihood of any chorioamnionitis, infection with *T. vaginalis* significantly increased the likelihood of any chorioamnionitis diagnosis (aPR 2.19, 95% CI 1.26-3.83).

**Conclusion:**

In this adolescent population with a high rate of PTB, in whom most received appropriate STI treatment, we did not find an association between STI during pregnancy and an increased rate of PTB. However, an infection with *T. vaginalis* was associated with an increased likelihood of chorioamnionitis. Early detection of STIs may prevent adverse pregnancy outcomes. Continued vigilance in STI screening during pregnancy, including consideration of universal *Trichomonas vaginalis* screening, is merited in this high-risk population.

## 1. Introduction

The national adolescent pregnancy rate and prevalence of sexually transmitted infections (STIs) in the United States (US) remain high compared with other industrialized countries [1–3]. Adolescents are especially vulnerable to acquiring STIs due to behavioral factors such as unprotected intercourse [4] and have a higher risk of adverse pregnancy outcomes, especially preterm deliveries [5–9]. These adverse pregnancy outcomes have been attributed to the physical immaturity of the uterus as well as the growth and nutritional status of the adolescent [10, 11]. Prior studies in primarily adult populations have shown associations between lower genital tract infection during pregnancy including *Chlamydia trachomatis*, *Neisseria gonorrheae*, and *Trichomonas vaginalis* and a higher risk of PTB and increased morbidity in the neonate [12–18].

The prevalence of *C. trachomatis*, *N. gonorrheae*, and *T. vaginalis* during pregnancy in adolescents is variable in the literature ranging from 7.5 to 37% for *C. trachomatis* and 1.5 to 10.4% for *N. gonorrheae* [4, 19, 20]. There is a wide range of prevalence, especially for *C. trachomatis*, in pregnant adolescents with the highest in urban center populations [17]. Less data about *T. vaginalis* exists, as it is not a reportable STI in the US. In a study of nonpregnant adolescents, the *T. vaginalis* prevalence was reported to be 14.4% [21]. Despite adolescents being at increased risk for preterm birth (PTB) and at increased risk for STIs, data are lacking that specifically address the impact of STIs on PTB and other infectious complications, such as chorioamnionitis, on the pregnant teenage population [20]. Thus, the objective of this study was to assess the prevalence of STIs during pregnancy and the associations between these STIs and PTB and chorioamnionitis among a cohort of pregnant adolescents.

## 2. Materials and Methods

### 2.1. Study Design and Population

**Figure 1:**
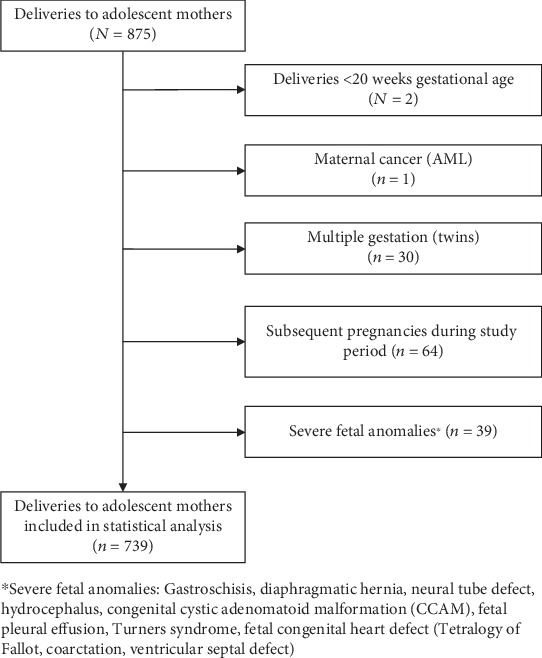
Study population derivation

We conducted a retrospective cohort study of adolescent pregnancies utilizing data from medical records from a large tertiary referral center in central Illinois. We included adolescents aged 13-19 years, who delivered between March 2010 and December 2014 at the health center. Exclusion criteria included adolescents who delivered prior to 20 0/7 weeks, multiple gestations, maternal cancer, and severe fetal anomalies defined as anomalies that—at least without treatment—were not compatible with life or led to severe morbidity (Figure 1). For adolescents with more than one delivery during the study period, we only included the first pregnancy. We obtained IRB approval from the hospital and University of Illinois (IRBNet ID 707363-5).

### 2.2. Data Sources, Abstraction, and Definitions

Medical records of adolescent patients with the ICD9 Code for delivery (V27.0-V27.9) were retrieved from the Electronic Medical Records System (EPIC) system. Each chart was manually reviewed by one of two physicians to confirm the accuracy of the electronic abstraction, particularly the diagnosis and treatment of STIs and chorioamnionitis during pregnancy. In addition, the first 40 charts were reviewed by both physicians to confirm interreviewer validation. After independent review and discussion of possible discrepancies, the remainder of the charts was reviewed by one physician. Prenatal records from private physicians' offices were also available as scanned documents in the EPIC system for all participants.

Routinely, STI testing was conducted at the first prenatal visit including *N. gonorrheae*, *C. trachomatis*, *T. vaginalis*, HIV, and syphilis. For most of the adolescents, the same testing was repeated later in pregnancy as recommended for high-risk groups by the Centers for Disease Control (CDC) [22]. Symptomatic patients received additional testing when indicated. Patients that tested positive had a test of cure after treatment. Only positive test results were included in the database. At this referral center and its associated clinics, *T. vaginalis* was primarily evaluated with the affirm VPIII test for vaginitis/vaginosis (Becton Dickinson, Franklin Lakes, NJ), and *N. gonorrhoeae* and *C. trachomatis* are assessed using the Probe TecTM Qx Amplified DNA Assay (Becton Dickinson, Franklin Lakes, NJ). Patients diagnosed with *N. gonorrhoeae*, *C. trachomatis*, or *T. vaginalis* were treated in accordance with published CDC treatment recommendations [23]. All patients diagnosed with *T. vaginalis* received a 2 gram oral dose of metronidazole. Patients diagnosed with *C. trachomatis* received a 1 gram oral dose of azithromycin and patients diagnosed with *N. gonorrhoeae* a combination of 250 mg intramuscular dose of ceftriaxone and 1 gram oral azithromycin. Partner treatments were provided to all patients with either receipt of a partner treatment pack, prescription of antibiotic, or recommended visit to the public health department.

For the purposes of this analysis, PTB was defined as delivery between 20 0/7 and 36 6/7 weeks of pregnancy [24]. The term chorioamnionitis is an imprecise diagnosis and refers to a heterogeneous group of conditions that may include inflammation and/or infection of the amnion and/or chorion [25]. We abstracted diagnoses for both clinical and histopathological chorioamnionitis from the EPIC medical record abstracted data and manual chart review. Clinical diagnosis of chorioamnionitis was made presumptively by the attending physician during labor in a woman with fever and other classical signs and symptoms such as maternal tachycardia, uterine tenderness, fetal tachycardia, or foul smelling amniotic fluid. Clinical diagnosis of chorioamnionitis from the chart was considered positive with diagnoses of ICD-9 658.4 and 762.7 included in the electronic medical record, or if criteria for diagnosis of chorioamnionitis were noted during chart review. Histopathologic chorioamnionitis was diagnosed when the placental pathology report noted chorioamnionitis, chorionitis, villitis, funisitis, or chorionic vasculitis [26, 27]. At this referral center, the placenta was sent for pathology for all patients with clinical suspicion for chorioamnionitis as well as in patients that delivered preterm. However, placental histology was not typically obtained in uneventful term deliveries.

### 2.3. Statistical Analyses

Demographic and reproductive history characteristics of participants with and without any STI during pregnancy were compared using chi-square (*Χ*^2^) for categorical variables and Wilcoxon's rank-sum test for continuous variables. We utilized Poisson regression with robust standard errors to estimate the prevalence ratio (PR) and 95% confidence intervals (CI) of the association between STI diagnosed during pregnancy and PTB and any chorioamnionitis (clinical or histopathological diagnosis) [28, 29]. Analyses were conducted for *C. trachomatis*, *N. gonorrhoeae*, and *T. vaginalis* separately and also for a combined STI exposure. The models were adjusted *a priori* for age (continuous), race (White, Black, and others including Asian, American Indian, Alaskan Native, multiracial, and others), and smoking (never, ever/quit, and current). For other potential confounding factors, including marital status, gravidity, and parity, we first conducted bivariate Poisson regression to estimate the association between these characteristics and PTB and any chorioamnionitis (clinical or histopathological) [28, 29]. Potential confounders that were associated with the outcomes with a *p* value < 0.2 were added one at a time to the multivariate model containing the *a priori* confounders. None of the potential confounding factors changed the association by more than 10% on the PR scale. Therefore, none were included in the adjusted models. Finally, for participants whose STI testing dates were available, we performed exploratory analyses of the association between trimester of each STI diagnosis and PTB and any chorioamnionitis to explore the timing of the infections on the adverse outcomes. Only the first positive STI test in a given pregnancy was included in these analyses.

The Midwest tertiary care center serves as a referral hospital for several counties within approximately a hundred-mile radius. The referrals of higher-risk pregnancies might inflate the PTB rate due to causes not associated with STI during pregnancy. To assess whether referral of high-risk adolescent pregnancies from other counties to the hospital affected the association between STI diagnosis and the pregnancy outcomes, we conducted a sensitivity analysis restricted to only deliveries among adolescents from the main catchment area; this includes the two counties closest to the hospital. Stata 13.0 was utilized for all analyses (StataCorp LP, College Station, Texas).

## 3. Results

Between March 2010 and December 2014, 875 babies were delivered at the institution to adolescent mothers between 13 and 19 years old. After applying exclusion criteria, 739 deliveries were eligible for analysis (Figure 1). The median age was 18 years (IQR:17-19). Half of the patients were White (55.8%, *n* = 411) and one-third Black (*n* = 268), and over 90% were single (*n* = 671) (Table 1). The prevalence of any STI during pregnancy was 16.5% (*n* = 122). Thirteen percent (13.1%, *n* = 97) of pregnant adolescents had a *C. trachomatis* diagnosis, 3.7% (*n* = 27) had *T. vaginalis*, and 3.1% (*n* = 23) had *N. gonorrhoeae.* Infection with any STI during pregnancy significantly differed by ethnicity, with Black adolescents having the highest prevalence (*p* ≤ 0.001) (Table 1). No patients were diagnosed with syphilis or HIV. The PTB rate was 18.8% (*n* = 139), and the rate of any chorioamnionitis was 15.2% (*n* = 112). The diagnosis of chorioamnionitis was made by clinical criteria in 22 patients (3.1%) and by placental pathology in 101 patients (26.5%). Only 5 of the participants had a second positive STI test, and all of which were positive for *C. trachomatis.*

### 3.1. Associations between STI and PTB and Chorioamnionitis

In unadjusted analysis, any STI during pregnancy was not associated with PTB (PR: 1.06, 95% CI 0.71-1.57) (Table 2). Similarly, *N. gonorrheae* (PR: 0.23, 95% CI 0.03-1.54), *C. trachomatis* (PR: 1.24, 95% CI 0.83-1.86), and *T. vaginalis* (PR: 1.19, 95% CI 0.58-2.45) during pregnancy were not associated with PTB. When adjusting for age, race, and smoking status, the associations remained similar and nonsignificant (Table 2).

In primary analysis, there were no significant associations between any STI, *N. gonorrheae*, or *C. trachomatis* and any chorioamnionitis (clinical or histopathological) (any STI-aPR: 1.33, 95% CI 0.88-2.02; *N. gonorrheae*-aPR: 1.14, 95% CI 0.47-2.80; and *C. trachomatis*-aPR: 1.31, 95% CI 0.83-2.07) (Table 2). In contrast, adolescents diagnosed with *T. vaginalis* during pregnancy had more than a twofold increased likelihood of chorioamnionitis compared to those not diagnosed with *T. vaginalis* (aPR: 2.19, 95% CI 1.26-3.83).

We conducted an exploratory analysis assessing the trimester of diagnosis of *C. trachomatis*, *T. vaginalis*, and *N. gonorrheae* with PTB and chorioamnionitis to ascertain whether the timing of infection might be a relevant factor (Table 3). *Chlamydia trachomatis* in the third trimester was associated with more than a twofold increased likelihood of PTB compared to participants without *C. trachomatis* during pregnancy (adjusted PR (aPR): 2.71, 95% CI 1.63-4.50). Neither *T. vaginalis* nor *N. gonorrheae* was associated with PTB in any trimester.

We did find some relationships between trimester of STI diagnosis and chorioamnionitis in the exploratory analysis. *T. vaginalis* diagnosed in the second trimester showed an increased likelihood of chorioamnionitis (aPR: 3.11, 95% CI 1.62-6.00), and diagnosis in the first trimester demonstrated a trend toward significance with chorioamnionitis (aPR: 2.29, 95% CI 0.91-5.76). *C. trachomatis* was not associated in any trimester with chorioamnionitis. For *N. gonorrheae*, we found a trend toward a significant association with chorioamnionitis when it was diagnosed in the third trimester (aPR: 2.77, 95% CI 0.96-8.02) (Table 3).

To further assess the association between *T. vaginalis* and chorioamnionitis, we also explored the method of chorioamnionitis diagnosis (clinical vs. histologic). When stratifying the main results by the method of chorioamnionitis diagnosis, there was a statistically significant association between *T. vaginalis* and chorioamnionitis diagnosed on pathology of the placenta (aPR: 1.90, 95% CI 1.10-3.26) and no association between *T. vaginalis* and clinical chorioamnionitis (aPR: 1.47, 95% CI 0.21-10.10).

### 3.2. Sensitivity Analysis

Adolescents from the hospital's immediate urban catchment area (*n* = 606), (the two counties surrounding the hospital with a population density approximately 100/km^2^), were different from outside referrals to the medical center (*n* = 133). When compared to adolescents living in other counties, the urban adolescents had lower levels of smoking (25.6% vs. 38.9%, *p* = 0.01) and were more frequently Black (41.7% vs. 12.0%, *p* < 0.001) and more likely to have a positive test for *C. trachomatis* (14.4% versus 7.5%, *p* = 0.03) or any positive STI during pregnancy (17.8% versus 10.5%, *p* = 0.04). The preterm rate for adolescents from referring counties was twice as high as the immediate catchment area (36.1% vs. 15.0, *p* < 0.001), but the chorioamnionitis rate was similar (15.7% vs. 12.8%). When restricting the analyses to the adolescent pregnancies from the hospital's immediate catchment area, associations between STI and PTB and chorioamnionitis were similar to the main results (Supplemental Tables [Supplementary-material supplementary-material-1]).

## 4. Discussion

Adolescents are considered a high-risk population for STIs and adverse pregnancy outcomes including PTB. In this large cohort of pregnant adolescents, 16.5% were diagnosed with an STI during pregnancy, nearly 20% experienced a PTB, and 15.2% had chorioamnionitis.

The CDC recommends screening of every pregnant woman for *N. gonorrheae* and *C. trachomatis* but not currently for *T. vaginalis*. In this cohort of adolescent pregnancies, the *C. trachomatis* prevalence of 13.1% and *N. gonorrheae* prevalence of 3.1% were elevated compared with 2.4-3.9% of *C. trachomatis* prevalence and 1.3% of *N. gonorrheae* prevalence among sexually active 14- to 19-year-old females estimated by NHANES [30, 31]. Even lower rates are recorded by the CDC (2017) in females aged 15–19 years with a *C. trachomatis* rate of 3.3% and a *N. gonorrheae* rate of 0.56%. In this cohort, the prevalence of *T. vaginalis* was 3.7%, but no adolescent NHANES data is available for comparison.

Data are mixed on the impact of *C. trachomatis* on PTB. Several studies show an increased risk of PTB or premature rupture of membranes in patients with *C. trachomatis* [16, 17, 19, 32, 33]. Other studies, including the current analysis, found no association between *C. trachomatis* and PTB. [12, 20]. However, in our exploratory analysis assessing trimester of infection, we found a nearly threefold increased likelihood of PTB among adolescents with *C. trachomatis* infection in the third trimester. However, these patients did not have a significant increase in chorioamnionitis diagnosis. The increased likelihood of PTB happened despite treatment in the third trimester. These results merit further study.

In our cohort, we did not find an association with PTB or chorioamnionitis and *N. gonorrheae*, as has been previously shown in large cohort studies that were not limited to adolescents [13]. However, we did see a trend toward the relationship of third trimester diagnosis of *N. gonorrheae* and chorioamnionitis. This difference in timing of diagnosis and subsequent treatment of *N. gonorrheae* deserves further study and may suggest that the inflammation did not have time to resolve in these patients. *N. gonorrheae* is associated with the presence of a robust response of neutrophils [34], which may partially explain this trend toward chorioamnionitis. While our data do not support an association of *N. gonorrheae* diagnosed during the third trimester with PTB, our overall PTB rate in this cohort was high relative to national rates which may make us less likely to detect a difference. Even after exclusion of adolescents referred to the hospital, the PTB rate of adolescents was still increased at 15% compared to the national average of 9.6% for all age groups [35].

Previous studies of *T. vaginalis* in pregnant patients have shown an increased risk for preterm premature rupture of membranes and preterm deliveries [18, 20, 36], but studies restricted to pregnant adolescents are scarce [20]. In this cohort, *T. vaginalis* during pregnancy was associated with more than two times increased likelihood of any chorioamnionitis diagnosis. This temporal association of *T. vaginalis* with chorioamnionitis in the second trimester with a trend toward an association when diagnosed in the first trimester is intriguing. This finding may suggest that despite early diagnosis and treatment, the risk of inflammation inciting chorioamnionitis does not resolve, with inflammation persisting in the choriodecidual lining. The fact that we did not detect an association of *T. vaginalis* and chorioamnionitis in the third trimester may suggest that a longer exposure time is more likely to result in chorioamnionitis. These findings would merit confirmation in other studies, as currently limited data exists on the timing of diagnosis of trichomoniasis and its association with chorioamnionitis. Despite this association of *T. vaginalis* with chorioamnionitis, we did not find an association with PTB.

This study had a number of strengths. With a sample size of 739 deliveries, it is one of the largest cohorts of pregnancy outcomes related to STIs in pregnant adolescents in the literature. Sensitive, reliable, and specific methods with DNA assays were used to detect *N. gonorrheae, C. trachomatis*, and most cases of *T. vaginalis*. Prior studies may have misclassified *T. vaginalis* infections by using less sensitive wet mount microscopy leading to underdiagnosis of trichomonas [37]. Lastly, we had data on the timing of infections which allowed exploratory analysis of the timing of the STI on the adverse pregnancy outcomes.

This study was subject to several limitations. First, medical records and the affirm VPIII test may not capture all STIs during pregnancy resulting in a likely underestimate of STI prevalence. The affirm test for diagnosis of *T. vaginalis infection* was used as standard of care in the study's clinical setting. This point of care test was chosen by clinical administration to ensure rapid treatment of *T. vaginalis*, bacterial vaginosis, and candida. Although, the sensitivity and specificity (63%, 99%, respectively) are lower than the Nucleic Acid Amplification test (NAAT) (95-100%, 95-100%) [38]. This test allowed for same day treatment and was guided by clinical care in a high-risk population and not the study investigators. In addition, we did not abstract data on bacterial vaginosis (BV) so could not adjust for this potentially concurrent infection. BV is associated with detection of *T. vaginalis, C. trachomatis*, and *N. gonorrheae* [12, 18, 39] and also with PTB, so we may have residual confounding by BV. Second, we are unable to determine which patients were diagnosed via STI screening versus testing done secondary to symptoms or preterm labor/contractions. We are thus unable to assess the effect of symptomatic versus asymptomatic infections on PTB or chorioamnionitis. Third, only the dates of positive STI tests were recorded so we were unable to report the overall proportion of young women who rescreened during pregnancy. Fourth, we did not differentiate between subtypes of preterm deliveries (spontaneous or medically indicated). In this population, the most common medical indication resulting in PTB was preeclampsia, and that diagnosis was evenly distributed in patients with and without STI. Furthermore, placental pathology was not routinely requested in uncomplicated term deliveries and was only available in 52% of deliveries, so our histologic evaluation of chorioamnionitis was not complete. There also remains a question if these results are generalizable to the entire adolescent population, particularly those aged 13-16, as the median age of the study was 18 years.

## 5. Conclusion

Any STI diagnosis during pregnancy was not associated with PTB or chorioamnionitis in this large cohort of adolescent pregnancies. However, adolescents infected with *T. vaginalis* during pregnancy had more than two times increased likelihood of chorioamnionitis diagnosis. Interestingly, *T. vaginalis* diagnosis in the early part of pregnancy was associated with chorioamnionitis. Whether a longer presence of *T. vaginalis* leads to inflammatory changes of the choriodecidual lining and also incites a more robust or longer lasting presence of inflammatory cells in the chorion and amnion, even after treatment, merits further study. Early screening and detection of Trichomoniasis might prevent an adverse pregnancy outcome as chorioamnionitis. Our data suggest that adolescents should also be screened for *T. vaginalis* in pregnancy. While this is not a current CDC recommendation, we found an association of *T. vaginalis* and the adverse pregnancy outcome of chorioamnionitis. This association was strongest when *T. vaginalis* was detected prior to the 3^rd^ trimester and suggests screening for *T. vaginalis* with a sensitive DNA-based test should be considered in pregnant adolescents early in pregnancy.

Overall, continued vigilance in STI screening during pregnancy is merited in this high-risk adolescent population.

## Figures and Tables

**Table 1:** Demographic and reproductive history characteristics and pregnancy outcomes for 739 study participants overall and by sexually transmitted infection status.

Characteristic^∗^	*N*	Overall		Any STI (*n* = 122)		No STI (*n* = 617)		*p* value
Demographic								
Age (median)	739	18	(17–19)	18	(17–19)	18	(17–19)	0.35
Age	739							0.66
13-15		36	(4.9)	5	(4.1)	31	(5.0)	
16-17		190	(25.7)	35	(28.7)	155	(25.1)	
18-19		513	(69.4)	82	(67.2)	431	(69.9)	
Marital status	738							<0.01
Single		671	(90.9)	120	(98.4)	551	(89.5)	
Married		60	(8.1)	2	(1.6)	58	(9.4)	
Divorced/separated		7	(1.0)	0	(0)	7	(1.1)	
Race	737							<0.001
White		411	(55.8)	44	(36.1)	367	(59.7)	
Black		268	(36.4)	72	(59.0)	196	(31.9)	
Other^†^		58	(7.9)	6	(4.9)	52	(8.5)	
Smoking status	727							0.96
Never		409	(56.3)	67	(55.4)	342	(56.4)	
Ever^‡^		115	(15.8)	19	(15.7)	96	(15.8)	
Yes		203	(27.9)	35	(28.9)	158	(27.7)	
Reproductive history								
Gravidity	739	1	(1)	1	(1)	1	(1)	0.79
Parity	739	0	(0-1)	0	(0-1)	0	(0-1)	0.63
Pregnancy outcomes								
Preeclampsia	739	45	(6.1)	6	(4.9)	39	(6.3)	0.55
Cesarean section	737	185	(25.1)	32	(26.2)	153	(24.9)	0.75
Gestational age at delivery	739							0.20
<34 weeks		74	(10.0)	17	(13.9)	57	(9.2)	
34-36 weeks		65	(8.8)	7	(5.7)	58	(9.4)	
37-38 weeks		178	(24.1)	33	(27.1)	145	(23.5)	
≥39 weeks		422	(57.1)	65	(53.3)	357	(57.9)	
Preterm birth	739	139	(18.8)	24	(19.7)	115	(18.6)	0.79
Any chorioamnionitis	738	112	(15.2)	24	(19.7)	88	(14.3)	0.13
Clinical chorioamnionitis	708	22	(3.1)	5	(4.4)	17	(2.9)	0.38
Chorioamnionitis by placental pathology	381	101	(26.5)	20	(33.3)	81	(25.2)	0.19

^∗^Results reported as *n* (%) or median (interquartile range). ^†^Including participants identifying as Asian, American Indian, American Native, multiracial, or others. ^‡^Including participants reporting quitting or no smoking during this pregnancy.

**Table 2:** Associations between sexually transmitted infections and preterm birth and chorioamnionitis.

Diagnosed STI	Preterm birth								Any chorioamnionitis							
	Yes (*N* = 139)		No (*N* = 600)		Unadjusted PR		Adjusted PR^∗^		Yes (*N* = 112)		No (*N* = 626)		Unadjusted PR		Adjusted PR^∗^	
	*n* (%)		*n* (%)		(95% CI)		(95% CI)		*n* (%)		*n* (%)		(95% CI)		(95% CI)	
Any STI	24	(17.3)	98	(16.3)	1.06	(0.71, 1.57)	1.09	(0.73, 1.62)	24	(21.4)	98	(15.7)	1.38	(0.92, 2.07)	1.33	(0.88, 2.02)
*N.* gonorrhoeae	1	(0.7)	22	(3.7)	0.23	(0.03, 1.54)	0.23	(0.03, 1.55)	4	(3.6)	19	(3.0)	1.15	(0.46, 2.85)	1.15	(0.47, 2.80)
*C. trachomatis*	22	(15.8)	75	(12.5)	1.24	(0.83, 1.86)	1.29	(0.86, 1.94)	19	(17.0)	78	(12.5)	1.35	(0.87, 2.11)	1.31	(0.83, 2.07)
*T. vaginalis*	6	(4.3)	21	(3.5)	1.19	(0.58, 2.45)	1.19	(0.58, 2.43)	9	(8.0)	18	(2.9)	2.30	(1.31, 4.04)	2.19	(1.26, 3.83)

Abbreviation: STI: sexually transmitted infection. ^∗^Adjusted *a priori* for age, race, and smoking status.

**Table 3:** Unadjusted and adjusted associations between trimester of STI diagnosis and preterm birth and any chorioamnionitis^†^.

	Preterm birth						Any chorioamnionitis					
	Yes *n* (%)	No *n* (%)	Unadjusted PR (95% CI)		Adjusted PR (95% CI)		Yes *n* (%)	No *n* (%)	Unadjusted PR (95% CI)		Adjusted PR (95% CI)	
*C. Trachomatis*												
	*N* = 137	*N* = 592					*N* = 111	*N* = 617				
Negative	117 (85.4)	525 (88.7)	Ref		Ref		93 (83.8)	548 (88.8)	Ref		Ref	
1^st^ trimester	7 (5.1)	29 (4.9)	1.07	(0.54, 2.12)	1.09	(0.55, 2.17)	7 (6.3)	29 (4.7)	1.34	(0.67, 2.68)	1.33	(0.67, 2.67)
2^nd^ trimester	4 (2.9)	27 (4.6)	0.71	(0.28, 1.79)	0.74	(0.29, 1.87)	6 (5.4)	25 (4.1)	1.33	(0.63, 2.81)	1.37	(0.56, 2.82)
3rd trimester	9 (6.6)	11 (1.9)	2.47	(1.48, 4.12)	2.71	(1.63, 4.50)	5 (4.5)	15 (2.4)	1.72	(0.79, 3.77)	1.48	(0.61, 3.62)
*N. Gonorrhoeae*												
	*N* = 139	*N* = 600					*N* = 112	*N* = 626				
Negative	138 (99.3)	578 (96.3)	Ref		Ref		108 (96.4)	607 (97.0)	Ref		Ref	
1^st^ trimester	0 (0)	7 (1.2)	—	—	—	—	1 (0.9)	6 (1.0)	0.95	(0.15, 5.86)	0.88	(0.15, 5.14)
2^nd^ trimester	0 (0)	11 (1.8)	—	—	—	—	1 (0.9)	10 (1.6)	0.60	(0.09, 3.94)	0.62	(0.10, 3.92)
3rd trimester	1 (0.7)	4 (0.7)	1.04	(0.18, 6.04)	1.06	(0.18, 6.09)	2 (1.8)	3 (0.5)	2.65	(0.89, 7.86)	2.77	(0.96, 8.02)
*T. vaginalis*												
	*N* = 139	*N* = 599					*N* = 112	*N* = 625				
Negative	133 (95.7)	579 (96.7)	Ref		Ref		103 (92.0)	608 (97.3)	Ref		Ref	
1^st^ trimester	3 (2.2)	6 (1.0)	1.78	(0.70, 4.56)	1.80	(0.71, 4.56)	3 (2.7)	6 (1.0)	2.30	(0.90, 5.90)	2.29	(0.91, 5.76)
2^nd^ trimester	2 (1.4)	8 (1.3)	1.07	(0.31, 3.74)	1.06	(0.30, 3.67)	5 (4.5)	5 (0.8)	3.45	(1.81, 6.58)	3.12	(1.62, 6.00)
3rd trimester	1 (0.7)	6 (1.0)	0.76	(0.12, 4.73)	0.77	(0.13, 4.66)	1 (0.9)	6 (1.0)	0.99	(0.16, 6.11)	0.95	(0.15, 6.11)

^†^Of the 739 adolescents, 97 (13.1%) adolescents had *C. trachomatis* diagnosed during their first pregnancy, 23 (3.1%) had *N. gonorrhoeae*, and 27 (3.7%) had *T. vaginalis*. 9 (1.2%) were coinfected by both *C. trachomatis* and *N. gonorrhoeae*, 2 (0.3%) by *C. trachomatis* and *T. vaginalis*, 8 (1.1%) by *C. trachomatis* and TV, and 3 (0.4%) by *C. trachomatis*, *N. gonorrhoeae*, and *T. vaginalis*. Five participants with *C. trachomatis* had a second positive *C. trachomatis* test later in pregnancy (0.7% of 739 in the cohort); second positive tests were not included in the analysis. Testing date was missing for 10 positive (10.3%) *C. trachomatis* results, and 1 test date was missing for a participant with *T. vaginalis* (3.7%).

## Data Availability

The dataset is only kept by the authors without any personal identifiers and available upon request. It is not publicly archived and therefore not available via hyperlinks.

## Supplementary Materials

Supplementary Materials Supplemental Tables 1 and 2 represent exploratory analyses comparing the counties of the primary catchment area to the referral counties to correct for referrals to the tertiary care hospital for reason of preterm delivery.
