# Supplementary material for: Influence of Sexually Transmitted Infections in Pregnant Adolescents on Preterm Birth and Chorioamnionitis
Source: Infect Dis Obstet Gynecol. 2020 Mar 25;2020:1908392. doi: 10.1155/2020/1908392 (PMC7132592; doi:10.1155/2020/1908392)
Supplement: Supplementary Materials — Supplemental Tables 1 and 2 represent exploratory analyses comparing the counties of the primary catchment area to the referral counties to correct for referrals to the tertiary care hospital for reason of preterm delivery. [file 1908392.f1.pdf]

Supplementary Tables

Manuscript Title: Influence of sexually transmitted infections in pregnant adolescents on preterm delivery and chorioamnionitis

Authors: Esther Fuchs, MD; Maggie Dwiggins, MD, Erica M Lokken MS, PhD; Jennifer A Unger, MD, MPH; Linda O Eckert, MD

Supplementary Table 1. Demographic and reproductive history characteristics, STI diagnosis, and pregnancy outcomes for 739 study participants – By County Type

Characteristic*	Total N	Primary Catchment Counties (n=606)		Referral Counties (n=133)		p-value
Demographic						
Age (Median)	739	18	(17-19)	18	(17-19)	0.45
Age	739					0.75
13-15		30	(5.0)	6	(4.5)	
16-17		159	(26.2)	31	(23.3)	
18-19		417	(68.8)	96	(72.2)	
Marital status	738					0.11
Single		556	(91.8)	115	(87.1)	
Married		46	(7.6)	14	(10.6)	
Divorced/separated		4	(0.7)	3	(2.3)	
Race	737					<0.001
White		303	(50.2)	108	(81.2)	
Black		252	(41.7)	16	(12.0)	
Other†		49	(8.1)	9	(6.8)	
Smoking status	727					<0.01
Never		350	(58.2)	59	(46.8)	
Ever‡		97	(16.1)	18	(14.3)	
Yes		154	(25.6)	49	(38.9)	
Reproductive History						
Gravidity	739	1	(1,1)	1	(1,1)	0.33
Parity	739	0	(1,1)	0	(0,1)	0.45
Sexually Transmitted Infections						
Any STI	739	108	(17.8)	14	(10.5)	0.04
N. gonorrhoeae	739	20	(3.3)	3	(2.2)	0.53
C. trachomatis	739	87	(14.4)	10	(7.5)	0.03
T. vaginalis	739	23	(3.8)	4	(3.0)	0.66
Pregnancy Outcomes						
Pre-eclampsia	739	30	(5.0)	15	(11.3)	<0.01
Cesarean section	737	147	(24.3)	38	(28.8)	0.28
Gestational age at delivery	739					<0.001
<34 weeks		40	(6.6)	34	(25.6)	
34-36 weeks		51	(8.4)	14	(10.5)	
37-38 weeks		138	(22.8)	40	(30.1)	

≥39 weeks		377	(62.2)	45	(33.8)	
Preterm birth	739	91	(15.0)	48	(36.1)	<0.001
Any Chorioamnionitis	738	95	(15.7)	17	(12.8)	0.40
Clinical Chorioamnionitis	708	21	(3.6)	1	(0.8)	0.09
Chorioamnionitis by Placental Pathology	381	85	(28.5)	16	(19.3)	0.09

Supplementary Table 2. Associations between sexually transmitted infections and preterm birth and chorioamnionitis – Primary Catchment Counties Only

Diagnosed STI	Preterm Birth								Any Chorioamnionitis							
	Yes (N=91) n (%)		No (N=515) n (%)		Unadjusted PR (95% CI)		Adjusted PR§ (95% CI)		Yes (N=95) n (%)		No (N=510) n (%)		Unadjusted PR (95% CI)		Adjusted PR (95% CI)	
**Any STI**	18	(19.8)	90	(17.5)	1.14	(0.71, 1.82)	1.13	(0.71, 1.81)	21	(22.1)	87	(17.1)	1.31	(0.84, 2.02)	1.31	(0.85, 2.02)
***N.* gonorrhoeae**	0	(0.0)	20	(3.9)	-	-	-	-	3	(3.2)	17	(3.3)	0.95	(0.33, 2.76)	0.93	(0.33, 2.64)
C. trachomatis	16	(17.6)	71	(13.8)	1.27	(0.78, 2.08)	1.27	(0.78, 2.08)	16	(16.8)	71	(13.9)	1.21	(0.74, 1.96)	1.22	(0.75, 1.99)
T. vaginalis	5	(5.5)	18	(3.8)	1.47	(0.66, 3.28)	1.37	(0.64, 2.93)	8	(8.4)	15	(2.9)	2.33	(1.29, 4.21)	2.19	(1.22, 3.92)

* Results reported as n(%) or median (inter-quartile range)
† Including participants identifying as Asian, American Indian, American Native, multiracial, or other
‡ Including participants reporting quitting or no smoking during this pregnancy
Abbreviation: STI-sexually transmitted infection

§ Adjusted *a priori* for age, race, and smoking status
